# Gender: shaping personality, lives and health of women in Pakistan

**DOI:** 10.1186/1472-6874-14-53

**Published:** 2014-04-01

**Authors:** Narjis Rizvi, Kausar S Khan, Babar T Shaikh

**Affiliations:** 1 Department of Community Health Sciences, Aga Khan University, Karachi, Pakistan

## Abstract

**Background:**

Gender norms determine the status of Pakistani women that influence their life including health. In Pakistan, the relationship between gender norms and health of women is crucial yet complex demanding further analysis. This paper: determines the reasons for reiteration of gender roles; describes the societal processes and mechanisms that reproduce and reinforce them; and identifies their repercussions on women’s personality, lives and health especially reproductive health.

**Methods:**

As part of a six-country study titled ‘Women’s Empowerment in Muslim Contexts’, semi-structured group discussions (n = 30) were conducted with women (n = 250) who were selected through snowballing from different age, ethnic and socio-economic categories. Discussion guidelines were used to collect participant’s perceptions about Pakistani women’s: characteristics, powers, aspirations, needs and responsibilities; circumstances these women live in such as opportunities, constraints and risks; and influence of these circumstances on their personality, lifestyle and health.

**Results:**

The society studied has constructed a *‘Model’* for women that consider them ‘*Objects*’ without rights and autonomy. Women’s subordination, a prerequisite to ensure compliance to the constructed model, is maintained through allocation of lesser resources, restrictions on mobility, seclusion norms and even violence in cases of resistance. The model determines women’s traits and responsibilities, and establishes parameters for what is legitimate for women, and these have implications for their personality, lifestyle and health, including their reproductive behaviours.

**Conclusion:**

There is a strong link between women’s autonomy, rights, and health. This demands a gender sensitive and a, right-based approach towards health. In addition to service delivery interventions, strategies are required to counter factors influencing health status and restricting access to and utilization of services. Improvement in women’s health is bound to have positive influences on their children and wider family’s health, education and livelihood; and in turn on a society’s health and economy.

## Background

Gender is a social construct that impacts both sexes [1]; women are however more vulnerable because of their subordinate status [2]. In most of the South Asian societies, women face discrimination because of some deeply rooted gender norms [3]. Pakistan is one of the developing South Asian countries with wide gender inequities [4]. Extensive gender gaps exist in education [5]; nutrition [6], health care [7] and employment [8]. Being signatory to international treaties such as Convention to Eliminate All Discrimination against Women, International Conference on Population and Development and Millennium Development Goals; the Pakistan government is obliged to achieve gender equality. Government’s efforts to fulfil its commitments are reflected to a certain extent in its policies on Health, Population and Women’s development, and programmes including Primary Health Care and Family Planning, and Maternal, New-born and Child Health. The country still, however, ranks low in gender indicators and its gender equality measurements are deteriorating [9].

Gender inequalities deprive women of their rights, autonomy and leadership [10]; hence affect their life’s prospects [11], specifically reproductive behaviours [12]. This causes delays in achieving social and health targets [13]. The four institutions of power (family, community, health care systems and the state) play an important role in determining the health status of women. Family traditions and customs govern the lives of women [14]. A locally conducted study in a metropolitan city of the country has shown that gender roles are repeated and culture and religion are used in socializing girls and boys to these roles [15]. However, it is yet unclear why gender roles are reiterated, which mechanisms and processes society use to reinforce and naturalize them and what implications they have on women’s personalities, lifestyles and health. The gender inequalities in the health care system have direct effects on the health care-seeking behaviors. Inappropriate or delayed health care-seeking could lead to undesirable health outcomes, high fertility, unwanted pregnancies, medical complications, and amplified susceptibility to future illnesses among women [16,17]. Survey reports and literature mainly provide information about married women that focuses primarily on reproductive health, particularly knowledge and practices related to family planning [18,19]. There is a dearth of information available on the lives of women as perceived by them with regard to their attributes, personality, desires, powers, responsibilities, risks, benefits, issues and problems.

The current paper therefore aims to: determine the reasons for reiteration of gender roles; describe the societal processes and mechanisms that reproduce and reinforce them; and identify their repercussions on women’s personality, lives and health especially reproductive health. To accomplish these aims the perception of Pakistani women were gathered about their lives in terms of: (1) Characteristics, powers, aspirations, needs and responsibilities; (2) Circumstances these women live in such as opportunities, constraints and risks; and (3) Influence of these circumstances on their personality, lifestyle and health.

## Methods

The current paper is based on the findings of a multi-country study titled “Women’s Empowerment in Muslim Context”, conducted in six Muslim countries, using Participatory Action Research. In Pakistan, the study included two squatter-settlements in Karachi, where the Community Health Sciences Department of the Aga Khan University has been providing primary health care services, since 1996. These two squatter settlements were selected to represent the urban–rural mix of the population; one is in the middle of the city representing the urban whereas the other is in the peri-urban area which is exactly similar to rural areas of the country. The residents of these squatter settlements are from different ethnic and socio-economic backgrounds.

The paper is developed on the findings of group discussions (n = 30) with women (n = 250) living in these areas. These women were from different: age groups such as adolescents, adults, middle aged, and elderly; socio-economic strata like lowest, lower-middle and higher-middle; and ethnic groups representing all the provinces of the country. The participants of each of these categories were invited separately to avoid the influence of dominant individuals on the submissive ones. Participants were selected purposefully using snowballing as the objective was to involve those who are more knowledgeable about the issue under research and conversant with the circumstances prevailing. In each discussion, a total of 8–10 women participated.

A discussion guide was developed to gather participants’ perceptions around main issues of Pakistani women as determined in the current literature and reports. The issues of Pakistani women included in the discussion guide were their; characteristics, powers, aspirations, needs and responsibilities; and circumstances these women live in such as opportunities, constraints and risks and the repercussions of these circumstances on women’s personality, lifestyle and health. Enquiries were made to understand the reasons for women’s compliance to societal norms. The guide however had neutral and open ended questions and probes to: provide opportunity to identify new, unknown and previously unidentified information; keep the discussions focused, uniform, objective and comprehensive; and avoid the influence of interviewer’s opinions on the participants.

These discussions were facilitated by two trained teams, both led by a sociologist. The discussions were recorded while notes were also taken by a note taker. The team also observed and noted any unusual verbal and non-verbal communication. Discussions were transcribed. These transcriptions were read several times to develop an understanding of the participants’ perception. Qualitative content analysis was done to describe the: manifest content, what the text says; and latent content, interpretation of the underlying meaning of the text. The text was divided into ‘meaning units’ that were condensed and labeled with a ‘code’ which were subsequently analyzed and grouped into categories and then themes were developed.

## Results

The results are based on participants’ (n = 250) perceptions about Pakistani women’s: (1) characteristics, powers, aspirations, needs and responsibilities; (2) Circumstances these women live in such as opportunities, constraints and risks; and (3) Rrepercussions of these circumstances on their personality, lifestyle and health.

### (1) Pakistani women: characteristics, powers, aspirations, needs and responsibilities

#### Characteristics

Women are not considered individuals and therefore have no identity and rights; a woman is a daughter, sister, wife or mother. They have to cover themselves from head to toe, remain within the house and comfort and obey those on whom their identities rely upon.

#### Powers

Women have no right to make decisions; all decisions ranging from type of dress to marriage are made by the men of women’s own family or the in-laws. From childhood, girls are informed, taught and trained to believe that only men who are physically powerful and hence mentally competent to make decisions; *‘She is counseled, and if this does not work, she is forced through threats and violence to believe that she is an object that has to be operated by a male family member’.* In cases where women challenge these patriarchal privileges and/or seek to enforce their rights, violence is used as a means to control them; hence setting examples that reduces the instances of resistance.

#### Aspirations

Women desire to make decisions, groom, be praised, loved, and get education and employment.

#### Needs

Girls need knowledge specifically about physical and physiological changes occurring around puberty and skills to protect themselves from all types of abuses.

#### Responsibilities

Women are responsible for fulfilling the ‘Reproductive Role’; bearing and rearing of children, household chores and social and religious responsibilities. Their respect is correlated to the extent of their compliance to this triple role; and a woman may be labeled immoral on challenging the role. A woman’s existence is linked to reproduction; *‘Woman is created (by God) for reproduction’*. Women are “respected” on becoming pregnant, considered “supreme” on delivering a male child, and their worth is closely linked to the number of children they reproduce; *‘A woman’s worth is gauged through “number of pregnancies” and “number of sons delivered”.*

### (2) Circumstances: opportunities, constraints and risks

#### Opportunities

Despite repeated probing, women did not report having any opportunity at all in the vicinity which could contribute to their development.

#### Constraints

Women mentioned several restrictions they face:

a) ***Lesser Allocation of Resources:*** Girls are consciously given lesser educational, employment and food resources.

b) ***Lack of Guidance and No Access to Information:*** Girls have neither any guidance from the parents nor they are allowed to access information, specially related to sexual and reproductive organs and physiology, sex and sexual relations under the misconception that such knowledge will enhance illegal (illegitimate) sexual relationships.

c) ***Restrictions on Mobility and Socialization:*** Under the pretext of protecting girls from sexual abuse, they are confined within homes and are not allowed to interact with anybody*.*

d) ***Prohibitions on Grooming:*** Women generally and girls specifically are not allowed to groom under the fear that men might get attracted by them. *“A girl is considered flirt and immoral if she does so”*.

e) ***Restrictions on Productive Work:*** Women are not allowed to work for money outside, since men feel that they will become more successful and independent.

#### Risks

Women reported several risks they encounter:

a) ***Early Marriage:*** Girls are married as early as possible after initiation of menses under the pretext that moulding into the reproductive role is easier at a younger age.

b) ***Reproductive Morbidity, Complications and Mortality:*** Early marriage, repeated pregnancies and use of abortion for contraception make women prone to reproductive tract diseases and sexually transmitted infections.

c) ***Violence:*** Girls and women are at risk of all types of violence. Vulnerability to abuse increases further because girls and women lack knowledge and skills to protect themselves.

### Repercussions of these circumstances on women’s personality, lifestyle and health

The circumstances women are living in influence their personality, lifestyle and health:

a) **Influence on Personality:** Lesser resource investment in girls results in an inferior status of women. Mobility restrictions isolate women socially, and make them lonely without support and guidance; *‘We have nobody to share our feelings and experiences with’.* Lack of autonomy causes hopelessness. Absence of knowledge about puberty makes girls ashamed of physical and sexual changes; *‘Menarche’ is an abrupt and upsetting incident for us’.* Lack of knowledge and skills to protect themselves from sexual harassment makes them fragile and weak. Early marriage and consequent loss of freedom worries them. Sexual harassment and being blamed for that causes continuous fear. Over work, lack of appreciation and exposure to all kinds of abuses leads to frustration that ends up in anxiety and stress and in extreme cases even depression. Consequently girls/women lack confidence, have low self-esteem, self-conscious, insecure, scared, fragile and anxious.

b) **Influence on Lifestyle:** Girls and women comply with the ‘Reproductive Role’ given to them. They stay at home as they are neither allowed nor prepared to interact or go out. Women’s economic contribution is constrained by lesser investment in their education and skill building along with mobility restrictions; however they still participate in income generation activities without jeopardizing the norms set for them. They are not empowered to make decisions and are dependent on the male members for every decision and action. Women are unable to manage the challenges of the external environment as they are not skilled to do so. Therefore they are confined in homes in a subordinate position; they obey orders and silently accept verbal, physical, social and mental abuse and only complain when their life is being threatened.

c) **Influence on Health:** Women’s health is affected in following ways:

*Malnutrition*

Except the post-delivery period in case of the male baby, when higher allowances are given so that boy can be breastfed, generally meager nutritional allocation and repeated pregnancies make them malnourished.

*Violence*

Girls/women experience wide variety of violence; it could be physical ranging from slapping to burning; verbal such as taunting, use of bad language; mental like threats of divorce and actual divorce; and sexual in the form of rape and incest.

*High Fertility*

Women repeatedly become pregnant to deliver as many children as possible preferably sons to become worthier. Child birth is even preferred over a woman’s life; *‘Family members insisted for continuation of pregnancy, even at the risk of the pregnant mother’s life. Mother died after delivering a baby boy. Family considered the death as God’s will’.*

*Low Contraceptive Use*

Women’s ability to enforce contraceptive use is very limited because of the unilateral power that their male partners/husbands exercise in fertility decisions. A woman is persuaded to continue bearing children until the family has at least one son; she sometimes delivers 7 or more daughters in order to accomplish the objective.

***‘Abortion’*** *as a method for Contraception*

In cases of pregnancy with a female foetus, a woman’s reproductive rights are often denied because her husband will coerce her to terminate the pregnancy; *‘If husband gets to know that the fetus is female, he asks for termination of pregnancy’.*

*Neglect and Mistreatment during Pregnancy*

In case of female fetus, pregnant woman is given less nutritious food and rest, not registered for antenatal care, neglected and even abused. *‘A woman had three daughters. She conceived fourth time. The in-laws, on hearing that the ultrasound examination has revealed that the fetus is female, physically abused the pregnant women to an extent that she started bleeding and died on her way to the hospital’*.

*Excessive Reproductive Morbidity and Mortality*

Women experience excessive reproductive morbidities and mortality because of nutritional deficiencies, repeated pregnancies, violence and use of abortion as a contraceptive method.

*Delay in Seeking Healthcare*

Unless serious, women neither discuss nor seek medical advice for sexual and reproductive morbidities, because discussion about sex, sexual organs and their problems is a taboo.

Delay in Accessing Health Facility

Except in life-threatening situation, the family does not take the woman to a healthcare facility, because taking a woman out of the house is considered disrespectful.

## Discussion

**Figure 1:**
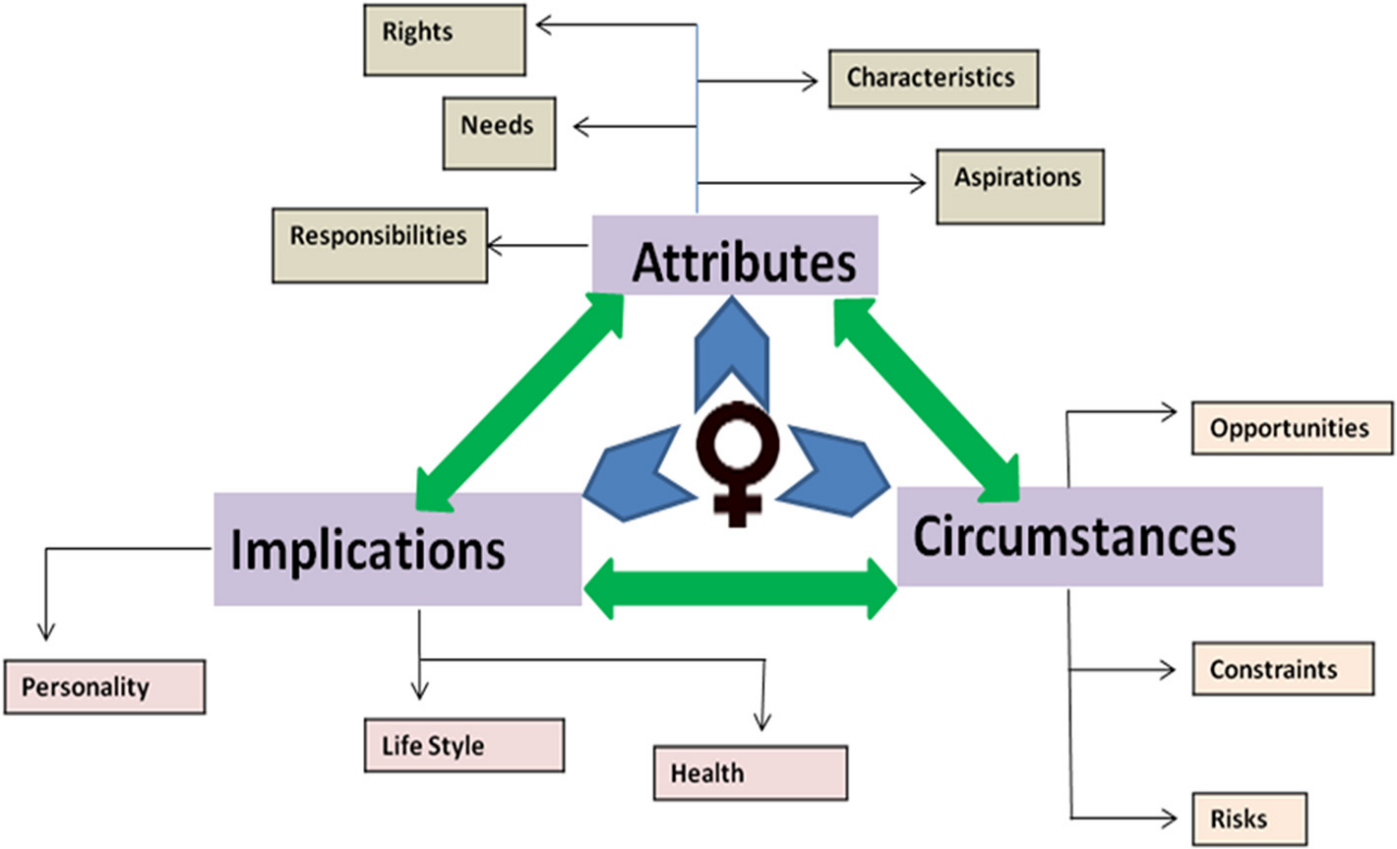
Depicting interlink among the attributes women possess, circumstances they live in and repercussions of these two on their personality, lifestyle and health; a vicious cycle.

**Figure 2:**
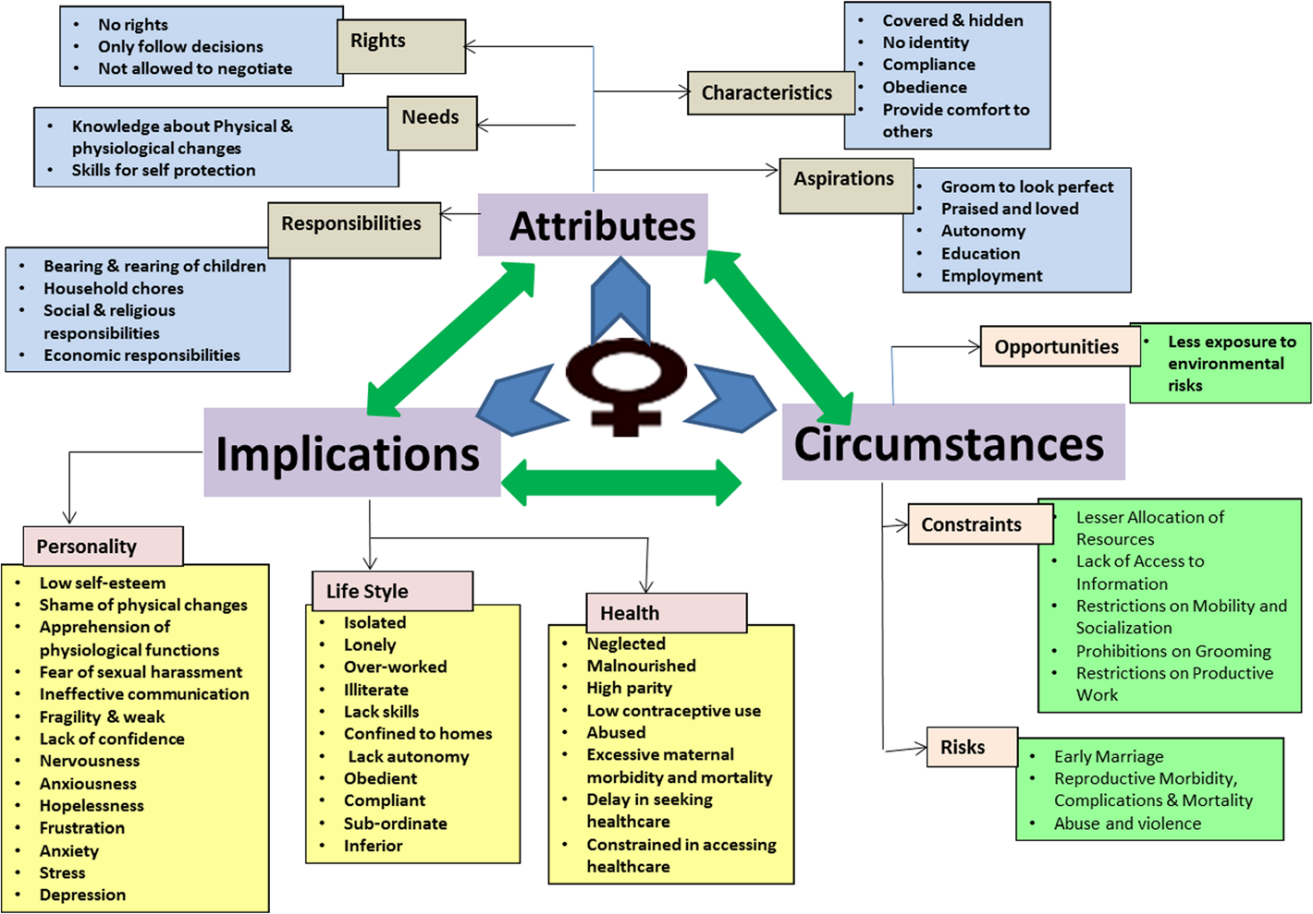
Depicting the potential role of determinants of attributes and circumstances and repercussions of these two on their personality, lifestyle and health in reiteration of gender roles and reinforcement of gender inequality.

Analysis of the perception of this sample of Pakistani women living in a poor urban settlement about their lives demonstrated that this society has constructed a model for them based on the principles that reproduction is a woman’s only responsibility, and the family honour is dependent upon her sexual chastity. An in-depth assessment of the information gathered from women revealed a strong interlink among the attributes women possess, circumstances they live in and repercussions of these two on their personality, lifestyle and health; a vicious cycle seems to be prevailing (Figure 1). A comprehensive understanding of the determinants of attributes, circumstances and repercussions of these two on their personality, lifestyle and health showed that all these factors reiterates gender roles and reinforces gender inequality (Figure 2).

The study identified that model constructed by the society determines the traits, responsibilities and parameters for a woman. The traits comprises of: covering of whole body; unconditional obedience to parent’s family before marriage and husband’s family after marriage; fulfillment of instructions without negotiation; home confinement with limited mobility; and expression of desires denied. The responsibilities include accomplishment of all household chores including stitching, bearing and rearing of children, care of ill and old, and participation in social and religious activities in the extended family. Parameters of “*Dos*” and “*Don’ts*” determine strictly the boundaries of a woman’s behaviour and actions, and thereby of her life; similar findings are reported from other studies in the country [20-22]. Consequently, the majority of the Pakistani women described in this paper are considered ‘*Objects’* without identity, rights and autonomy. Being signatory to the international treaties and commitments for promotion of individual human rights (United Nations, 1948; CEDAW, 1979; ICPD, 1994, UNIFEM, 1998), Pakistan needs to make its own commitments effective, and find strategies to ensure women’s access to basic rights such as autonomy, free mobility and expression of desires. Provision of these rights enhances self-worth, dignity and status and enables individuals’ capacity to negotiate and address injustices [23]. Once empowered, Pakistani women will be able to challenge the ‘Model’ foisted on them and actively participate in developing traits and parameters based on human rights principles that acknowledge women’s individuality.

The study revealed that through low investment in girl’s health and education [7,24], family and society reproduce and maintain women’s systematic subordination as being practiced for decades [20,21,25]. Having inferior status, women are compelled to follow the socially constructed model that disempowers them into surrendering their own abilities to take decisions; forces them to abide by the pre-established norms that restrict mobility, controls their social interactions and limits access to education and information; pushes them into early marriage and violence; and excludes them from the larger society of which they are a significant part. This calls for gender sensitive budgetary allocation in every sector but more importantly in health and education, so that women can get social and economic gains [26] in order to raise their status.

We found that society with the aim to preserve women’s chastity imposes certain norms like mobility restriction, social quarantine and prohibition on accessing information about sexual and reproductive issues [27], even when needed [28,29]. It is upsetting to note, however, that such customs have not succeeded in protecting women since sexual abuse is on the rise [30-32]. On the other hand such customs negatively influence a woman’s personality and social relations. As a person she lacks confidence, self-esteem and motivation that leads to powerlessness, stress, anxiety and depression; a finding reported in studies within Pakistan [12] and South Asian countries [2,20,33,34]. Socially, she is unable to interact and communicate effectively and manage the challenges of external environment appropriately [27,35-37]. This situation demands provision of relevant knowledge and skills to girls and the women that can inculcate confidence and self-reliance, and equip them with abilities to address circumstances they encounter. Two interventions that have been proved to be effective are Basic Life Skills [38] that can be introduced at every institution such as home, schools, madrassas (Religious schools), and use of electronic media such as TV, radio and cell-phone text messages [39].

The study further identified that women’s reproductive health is influenced by the ‘*Reproductive Role*’ which is one of the traits of the model society has strategized for them. The ‘*Reproductive Role*’ disallows women to: regulate their fertility; discuss sexual and reproductive health issues; and seek health care even when crucial such as during pre-natal, natal and post-natal periods. This finding is validated through national surveys reporting low utilization of reproductive health services by Pakistani women like contraception, tetanus toxoid vaccination, antenatal care and delivery by skilled birth attendant [7,24,40]. Consequently, sexual and reproductive morbidities remain unreported, untreated and many a times become intensive, complicated and fatal for mother and the child [41]. For decades, the same webs of causative factors in the country are responsible for not allowing women to seek skilled healthcare though [42,43], significant underlying determinants however are limited autonomy [11,44,45] and gender inequality [46]. Strategic strategies for enhancement of gender equity [47], women’s autonomy and status of girl child are vital [34]. However, during the transition phase, the reproductive health of women with restricted mobility can be improved by introducing operational interventions such as involvement of men to influence women’s reproductive behaviour [45,48] and delivery of skilled healthcare at the door-step through community-based health workers [49-51] and as a long term measure, deploying enough female health staff at the health facilities [52]. Similarly, family planning programs must look into gender dynamics in the society and even at the community level to ensure an equal access to contraceptives by men and women [53].

The robust methodology and rigorous analysis provides us confidence, though, that the findings of this study can be used to explain the experiences of other Pakistani women who are in comparable situations. However it should be remembered that this was not a quantitative study where results are statistically generalizable to the whole country. Although to eliminate interviewer’s bias a pre-designed discussion guideline was used without leading questions, however there could still be some interviewer’s influence on the responses.

## Conclusion

The model constructed by studied community considers women ‘*objects*’ without rights and autonomy. Compliance to this model in many cases is ensured by maintaining women’s subordination which is achieved through inadequate allocation of resources, mobility restrictions, and limited access to information, seclusion norms and even violence in cases of resistance. This disenfranchised model regulates women’s traits and responsibilities, and establishes parameters for their desires, behaviour and practices; all of these influence their personality and lifestyle, hence their health. More alarming is the contributing link existing between the attributes promoted by the constructed model and the circumstances created for women to adopt the model; a vicious cycle reiterating gender roles and reinforcing gender inequality (Figure 1).

As a consequence of this state of affairs, many Pakistani women in similar circumstances are illiterate; ill-informed; lack confidence and self-worth; disempowered; prone to violence; at risk of physical (reproductive and mental) illnesses; and unable to discuss health issues and seek healthcare when needed. The link between health, women’s autonomy, rights and status identified half a century ago [54] need urgent attention and actions focusing on gender sensitive and right-based approach are required. Concurrently, the conventional intervention-based health package needs to introduce strategies that counter socio-cultural factors influencing health status and outcomes [55], so that unacceptably high maternal mortality and morbidity can be reduced [56-58]. In this regard the determinants of each of the factors of the constructed model (Figure 2) can be utilized for development of strategies and interventions that can promote gender equality; hence improve women’s life including health. A three-pronged strategy is proposed: (1) advocacy efforts to convince policy makers for development of gender sensitive policies; (2) designing of programs, interventions and services keeping in view socio-cultural factors influencing health and healthcare services; and (3) behaviour and attitudinal change at individual, family and community levels to create an enabling environment where women can negotiate to exercise their right to health, and challenge their institutionalized neglect.

Women’s experiences of pregnancy and childbirth exert influences far beyond their own health, on their children and wider family’s health, education and wealth; indeed society health and economy [59,60]. The notion of equity, oft-associated with access, ought to be translated into equal utilization for equal need and equal quality of care for women. Strategies for advancing women’s strategic interests, along with meeting their practical needs would lay the foundation for women’s empowerment so that they could challenge and change the local gender systems.

## Competing interest

The authors declare that they have no competing interests.

## Authors’ contributions

NR made substantial contributions to the design of the study and acquisition of data, analysed and interpreted data and drafted the manuscript. KSK conceptualized and designed the study, reviewed the manuscript critically and made changes in the content. BTS made substantial contributions to the interpretation of data, critically reviewed the manuscript and made important intellectual additions to the content. All authors read and approved the final manuscript.

## Pre-publication history

The pre-publication history for this paper can be accessed here:

http://www.biomedcentral.com/1472-6874/14/53/prepub
